# Osteonecrosis Mandibular Extended to Bisphosphonates: A Very Rare Extensive Case

**DOI:** 10.7759/cureus.7428

**Published:** 2020-03-26

**Authors:** Lahcen Khalfi, Abibou Ndiaye, Wilfried Chabi, Mohammed Kamal Fiqhi, Karim El Khatib

**Affiliations:** 1 Plastic and Maxillo-Facial Surgery, Mohammed V Teaching Armed Forces Hospital, Rabat, MAR; 2 Surgery, Mohammed V Teaching Armed Forces Hospital, Rabat, MAR

**Keywords:** iatrogenic, disease mandibular, biphosphonate, zoledronate, complications, large necrosis, resection, surgery

## Abstract

After the first report of bisphosphonate-related osteonecrosis of the jaw (BRONJ) in 2003, it has increased significantly since then. We report a very rare extensive case never seen before in our experience of bone exposure with necrosis reaching the mandibular inferior border. Although the treatment modalities are not yet established, most researchers have recommended conservative approaches. The surgery was to be as conservative as possible, with a resection of the mandibular range followed by reconstruction using titanium plate with space maintainer. The authors would like to share their approach, management, and awareness.

## Introduction

After the first report of bisphosphonate-related osteonecrosis of the jaw (BRONJ) in 2003 [1-4], the American Association of Oral and Maxillofacial Surgeons (AAOMS) defined BRONJ in their 2009 position paper as “necrotic bone exposure in the maxillofacial region lasting for more than 8 weeks in patients with previous or current administration of bisphosphonate (BP) and with no history of radiation therapy” [4-5]. In the latest position paper in 2014, AAOMS modified the term into medication-related osteonecrosis of the jaw (MRONJ) to emphasize the role of drugs causing osteonecrosis of the jaw (ONJ) [4, 6]. Although the pathophysiology of jaw necrosis due to drug use is not yet fully understood, significant inhibition of bone remodeling based on the drug’s pharmacological effect has been considered to be the main cause [4]. Very recently, new medications have also been associated with ONJ [6]. We report a very rare extensive case of mandibular necrosis due to the BP therapy with review of the literature.

## Case presentation

**Figure 1:**
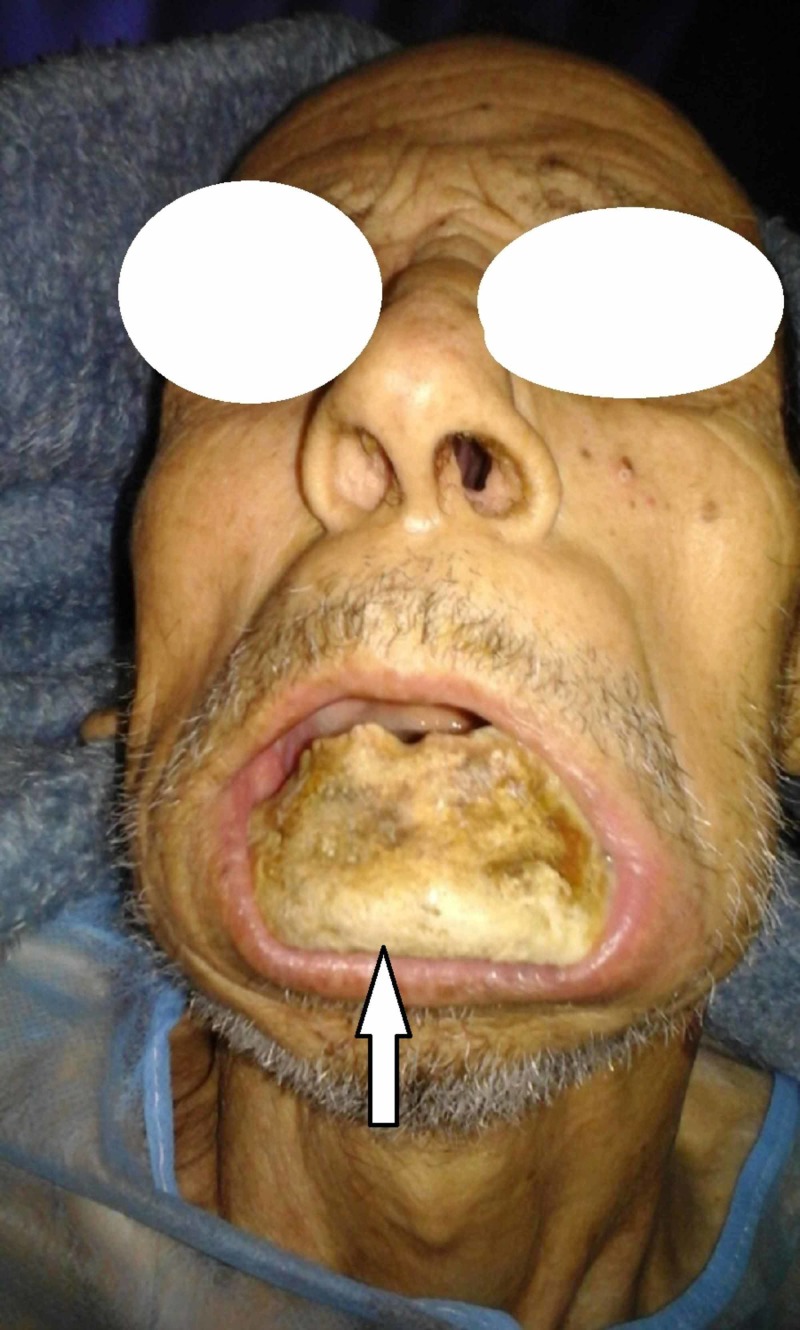
Patient's frontal view with bone of the jaw exposed.

**Figure 2:**
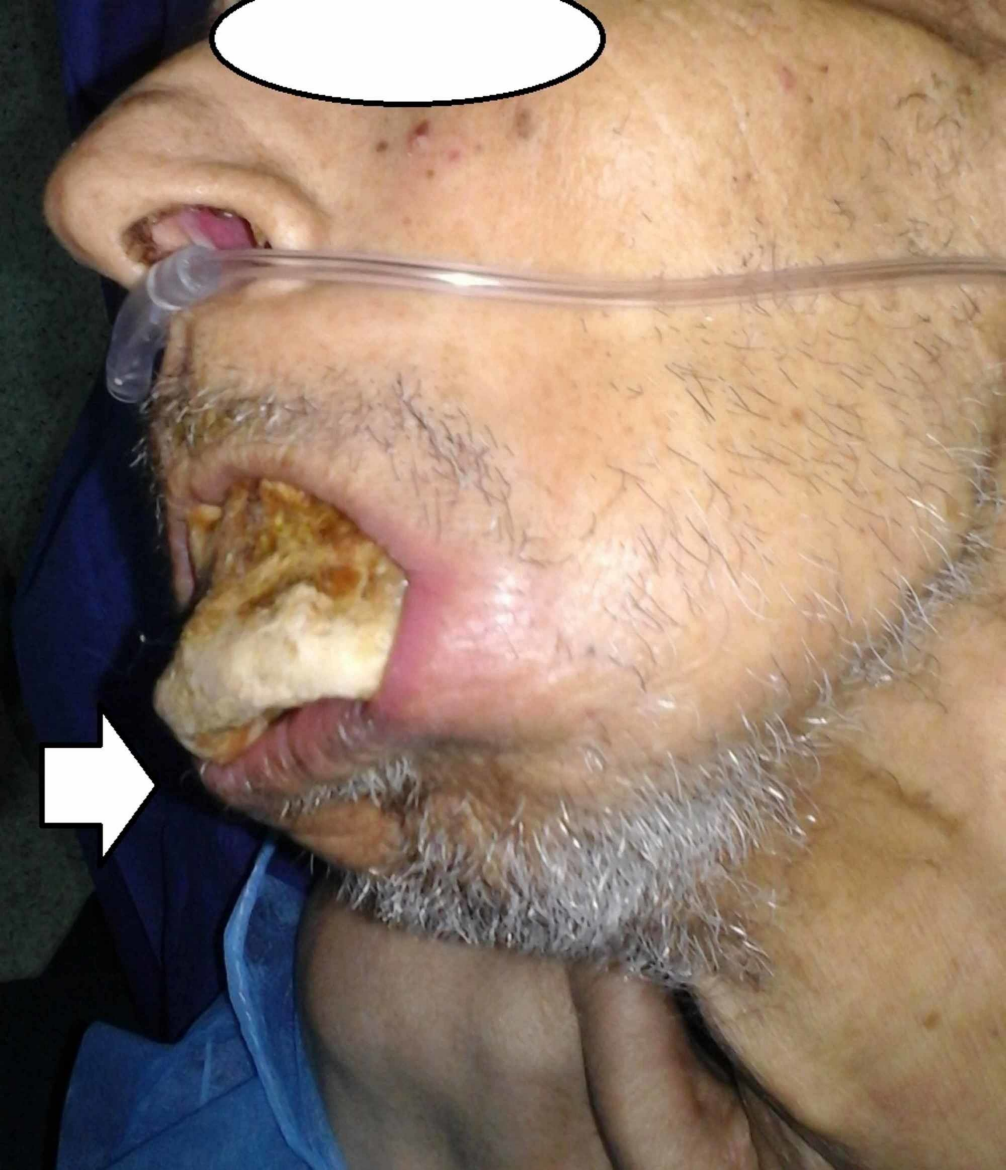
Patient's lateral view with ''degloving'' bone of the jaw.

**Figure 3:**
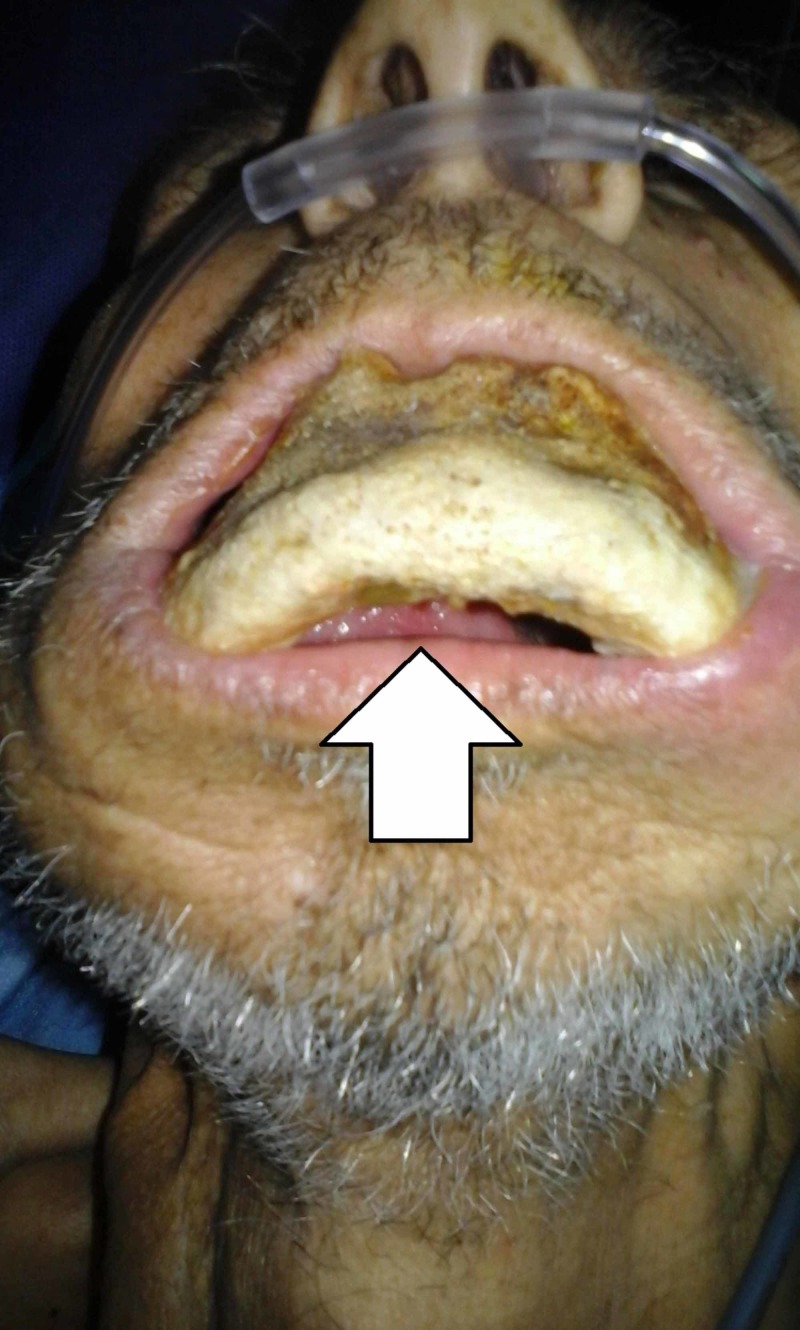
Patient's inferior view of communication to the oral cavity with ambient environment.

 A 68-year-old patient was referred to us in emergency condition by a hematologist for restricted opening of the mouth with bone exposure and loss of eating pleasure. In his history, the patient was followed in clinical hematology for multiple myeloma treated with eight CTD cures (cyclophosphamide, thalidomide, dexamethasone) in addition to monthly zoledronate (Zometa®, Novartis Pharma Maroc group, Morocco) cures years ago. We found the patient quick-tempered, grumpy, and agitated with no functional autonomy; he was in wheelchair in a bad general state of condition. Facial examination revealed bone denudation reaching the mandibular inferior edge. The oral examination showed any possibility of opening his mouth with a total toothless and bone exposure over the alveolar bone (Figures 1-3). 

**Figure 4:**
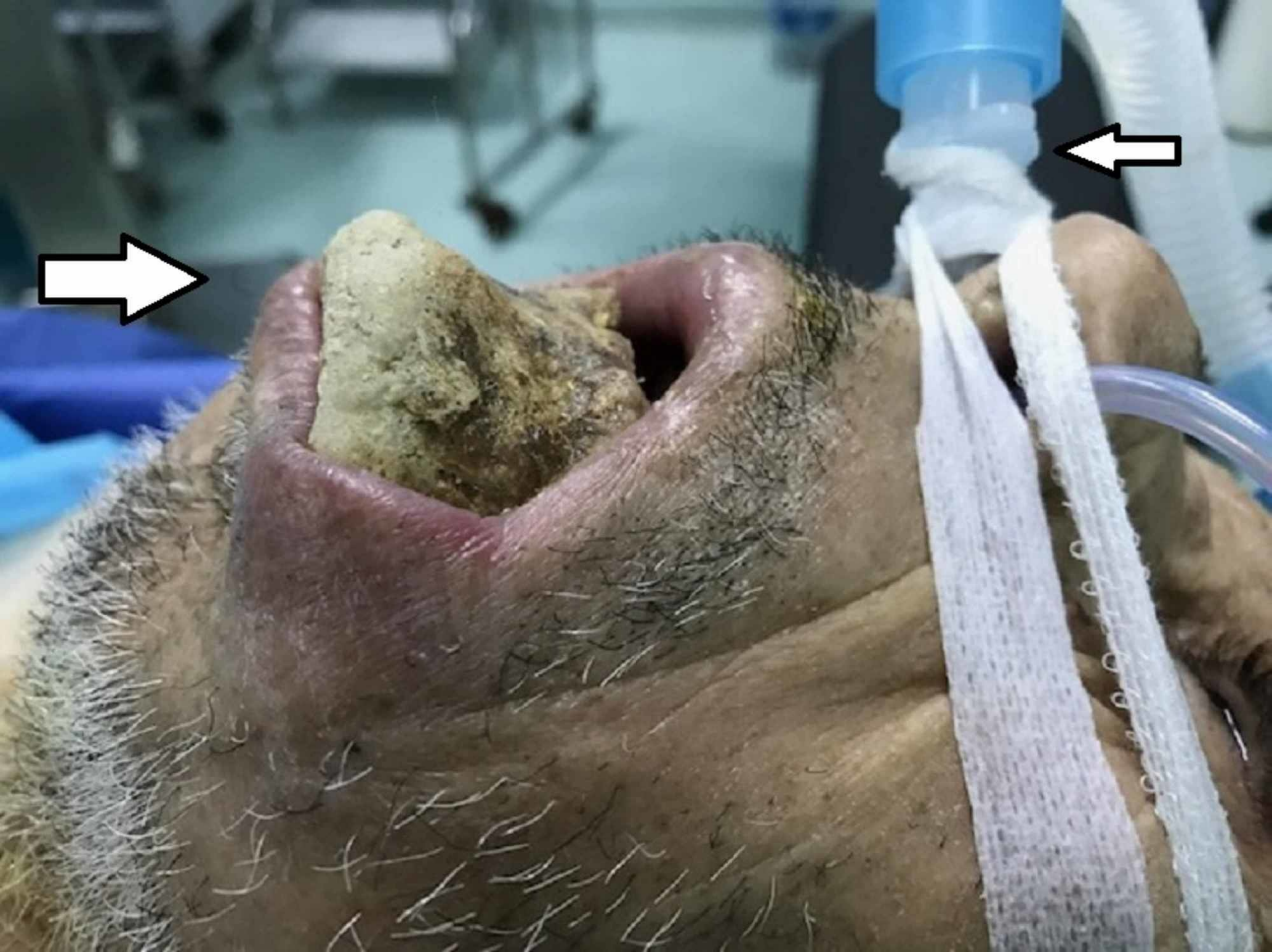
Per-operatoire view installation.

**Figure 5:**
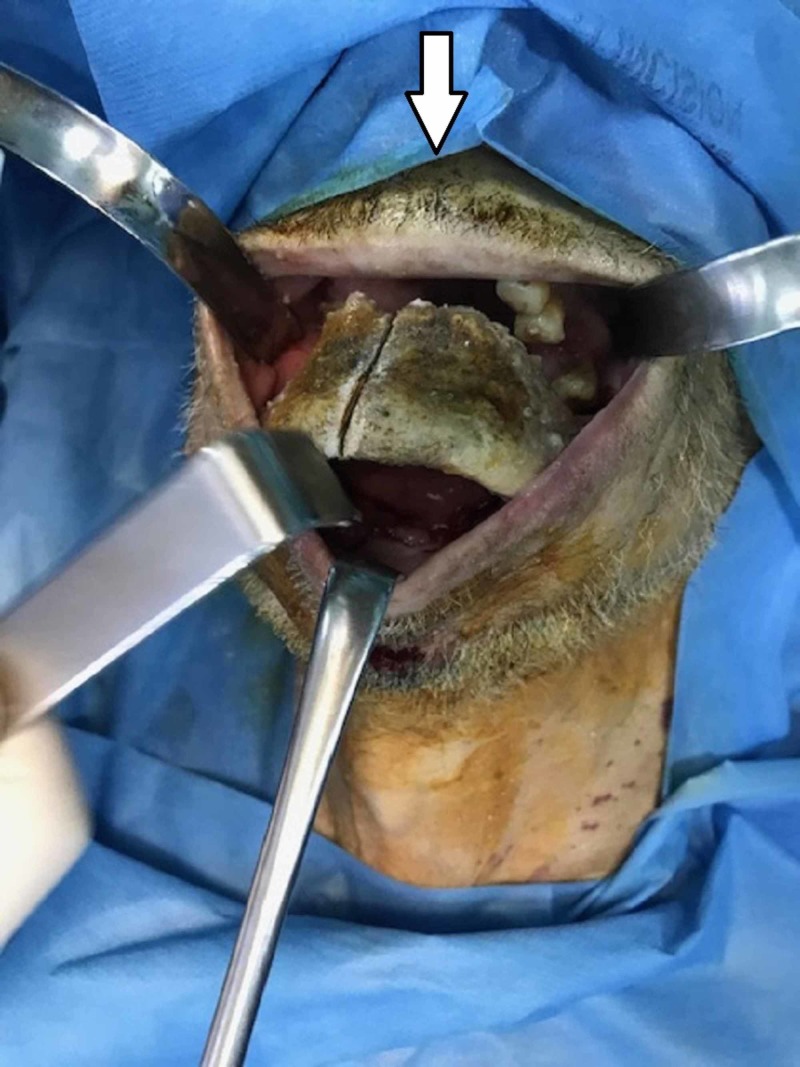
Per-operatoire view of the resection.

**Figure 6:**
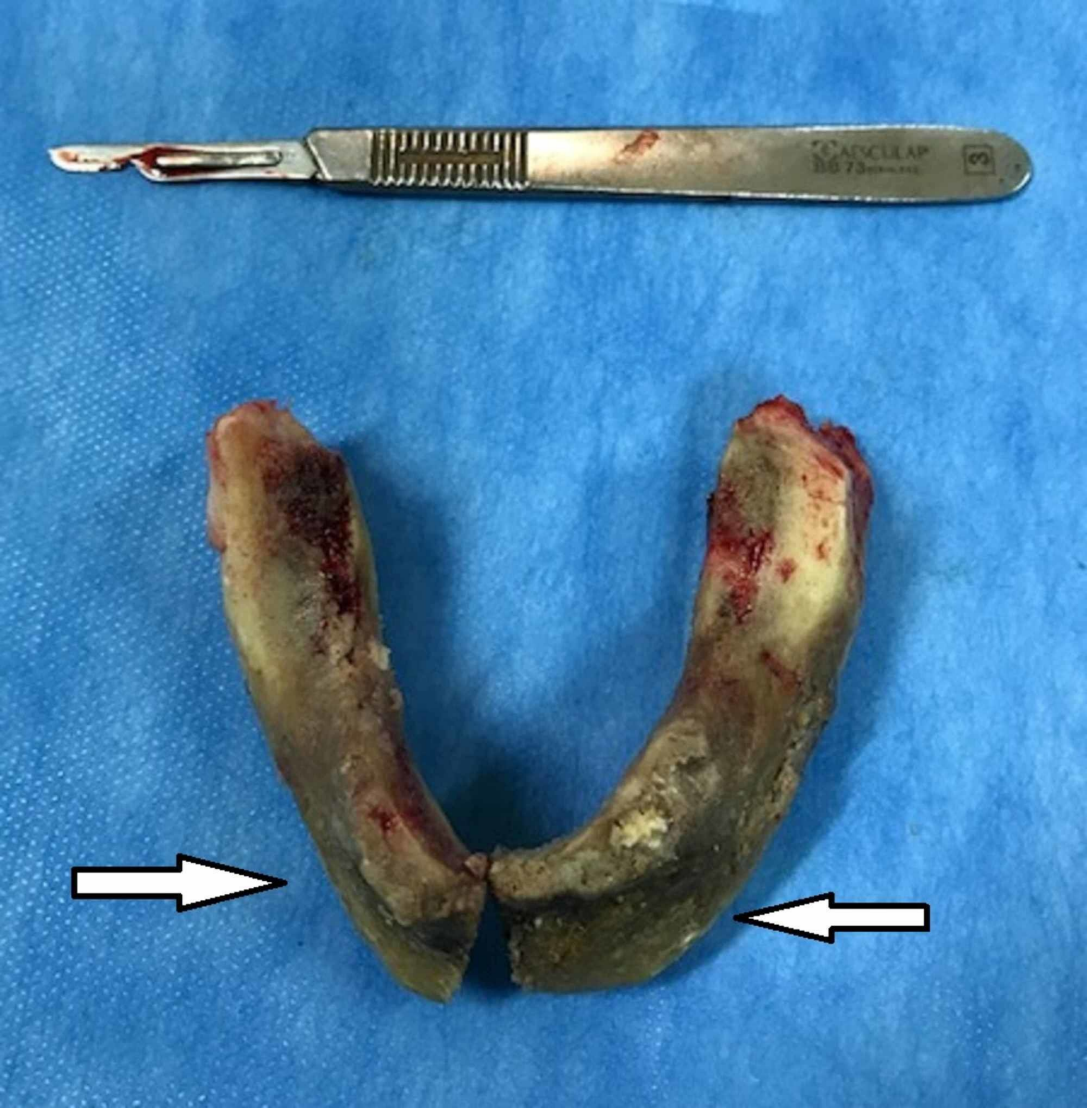
Resection piece view with extensive necrotic body of mandibular bone.

The medical history taking did not report any extraction of tooth before. After the final review, the patient received medical treatment of antibiotic therapy amoxicillin/clavulanic acid 1G/8H for suspicion of septic mandible osteonecrosis. The patient was prepared to cooperate but it failed and he refused to do any radiological examination (scan and orthopantomogram-OTP). As differential diagnosis in other forms of osteonecrosis, such as osteoradionecrosis and osteomyelitis of jaw, BRONJ is retained in the absence of radiotherapy and the duration of follow-up without purulent discharge. Later, the patient was transferred to resuscitation for pre-condition, with a filling protocol followed by a pre-operative checkup. We realized a segmental resection with subtotal mandibulectomy under general anesthesia. It is followed by the reconstruction of the entire body using titanium plate with space maintainer (Figures 4-6).

**Figure 7:**
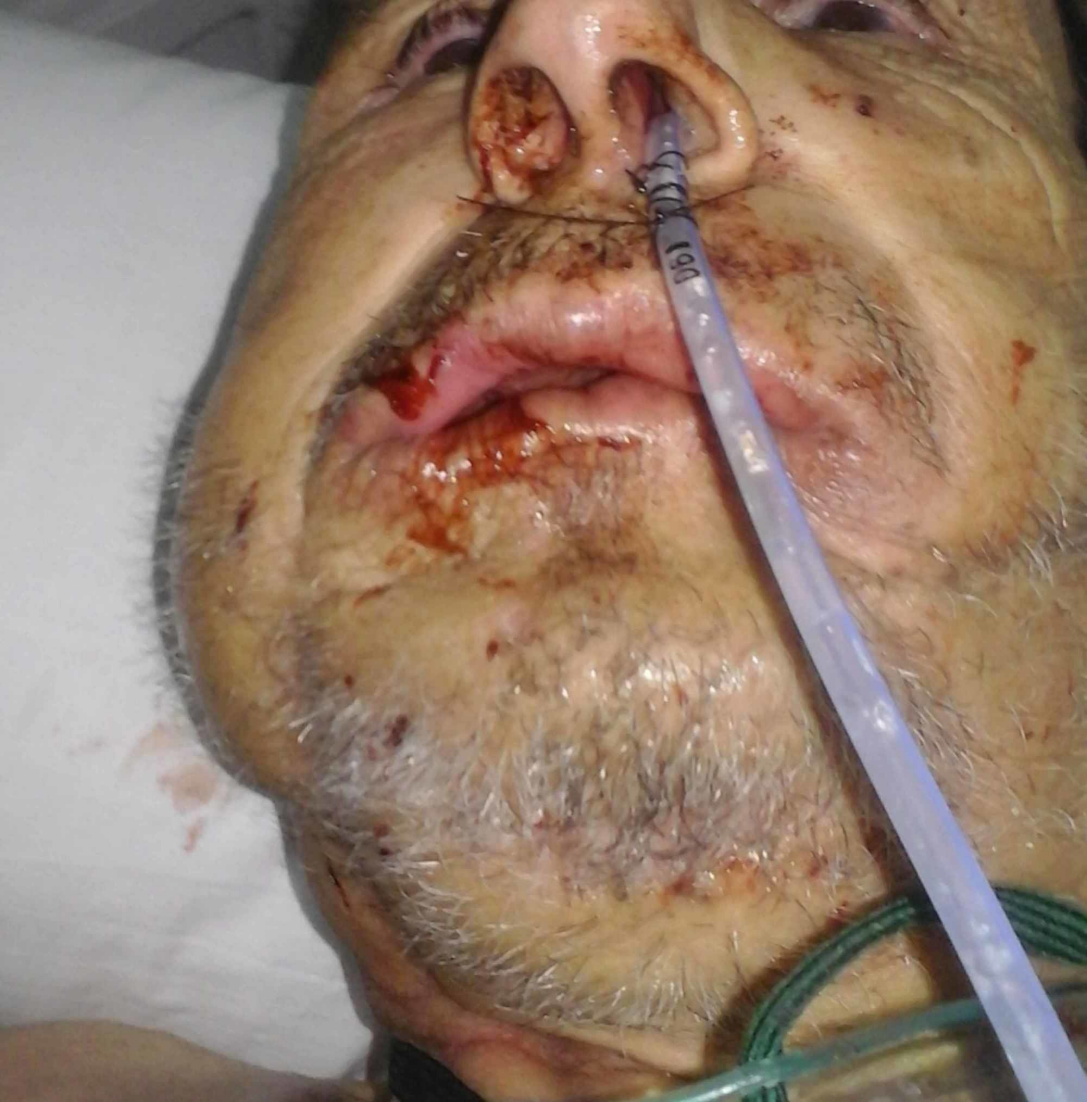
Postoperative view.

 In post-op care, a hyperprosthetic enteral nutrition was introduced by setting a gastric feeding tube. The diagnosis of osteonecrosis has been confirmed by histopathological study. After surgery, the patient’s condition improved well, allowing for the resumption of oral, liquid, semi-liquid, and mixed feeding (Figure 7).

**Figure 8:**
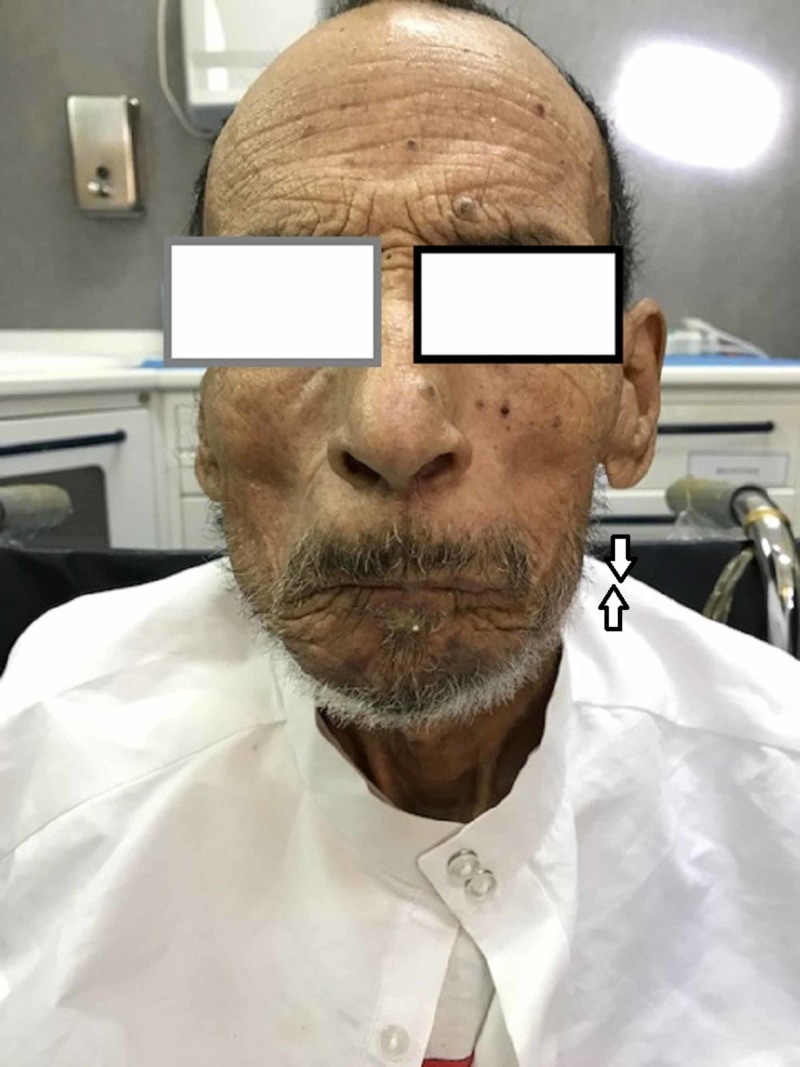
Control view: functional jaw/closed mouth.

**Figure 9:**
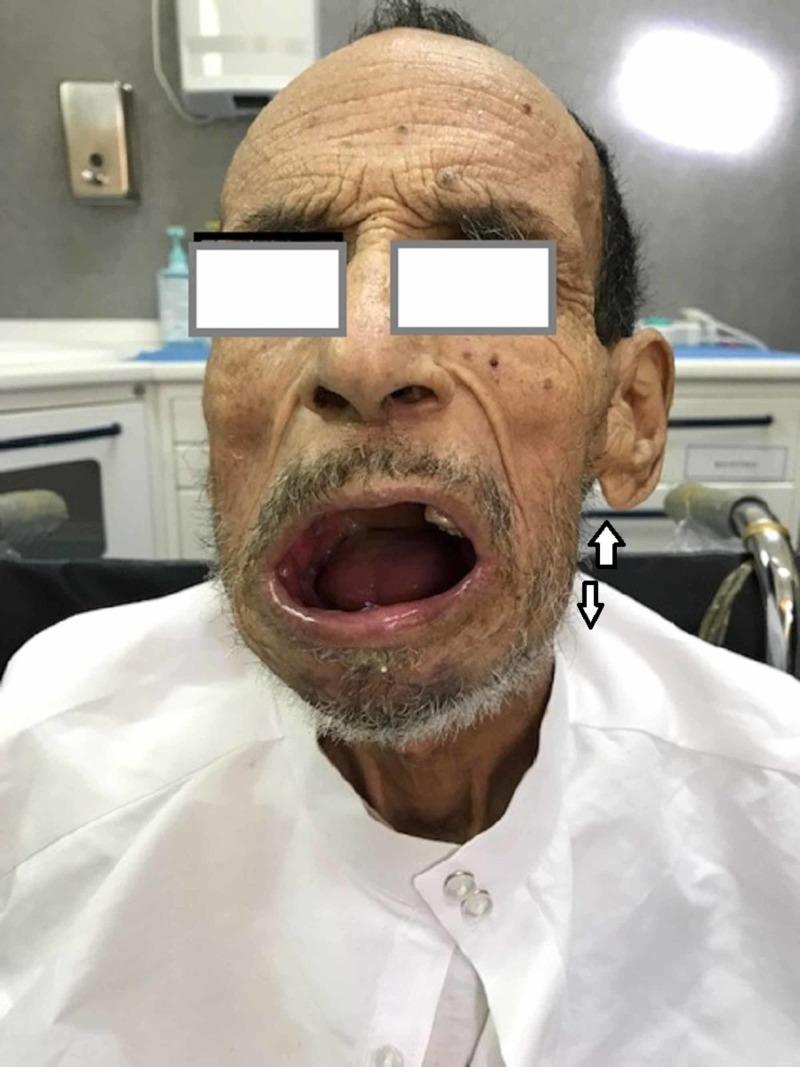
Control view: functional jaw/open mouth.

The periodical checks carried out show a relatively good state of health of the patient, without exposure of material (Figures 8-9). 

.

## Discussion

After the first report of BRONJ in 2003 [1-3], the AAOMS modified the term into MRONJ to emphasize the role of drugs causing ONJ [4, 6]. Additional studies have indicated that ONJ can be caused by single agent of corticosteroids, methotrexate, and recreational or illicit drugs such as cocaine, amphetamine, and methamphetamine [7]. However, local factors in the oral cavity, despite being absent from the formal definition of MRONJ, also increase risk and play a crucial role in the disease process [8-10]. This complication often occurs in patients at risk such as the age, immune status, nutritional status, and Karnofsky’s "performance status" [11-13]. In our case, the ONJ appears to be linked to the zoledronate (Zometa) treatment with potential promoters of osteonecrosis as chemotherapy, corticotherapy, and poor oral status. But the extent of bone dehiscence could be explained by the contamination of the affected bone, from the oral flora involved in the extension of the process of necrosis [14]. Bisphosphonates are inhibitors of osteoclastic resorption used to treat bone-remodeling disturbances and, therefore, used to prevent bone loss and preserve the integrity of the bone structure [5, 14]. They are indicated in osteoporosis, Paget’s disease, multiple myeloma like our patient and bone metastases from solid tumors and in malignant hypercalcaemia as well [12-14]. Only lesions of maxilla-mandibular necrosis are reported in the context of BP intake [12] and mandibule is reached twice than the maxillary probably because of the terminal vascularization [15]. As most often described, the exposure of necrotic bone occurs after gum mucosal ulceration secondary to surgery, dental removal, trauma, or apparently spontaneous disorders [14-15]. The medical history taking did not report any extraction of tooth or triggering factors for our patient. To classify the bone exposure, we proceed a review of the staging system in the literature and has found many criteria for BRONJ cases classification. In 2007 McMahon et al. describe as Stage 6 of 6 more than 4 cm of exposed necrotic bone with cortical fenestration and infection with severe and constant jaw pain [6]. In 2017 Yoneda et al. by the Japanese Committee on Osteonecrosis of the jaw describe as Stage 3 of 3 a bone exposure with necrosis over the alveolar bone reaching the mandibular inferior border or mandibular ramus [6, 16]. These descriptions appear to be closer to our case at least in view of bone exposure with necrosis over the alveolar bone reaching the mandibular inferior border. BRONJ usually demands a multi-disciplinary approach in the diagnosis, treatment, and prevention [4]. Although the treatment modalities are not established, most researchers have recommended conservative approaches [4], which management has been based on a staging system created by AAOMS in 2007 according to the severity of its signs and symptoms [5]. Surgical modalities range from conservative (debridement, bone curettage, and sequestrectomy) to radical (marginal/segmental resection), and increasing evidence suggests that surgical treatment is more effective than nonsurgical treatment [7]. The surgical resection of necrotic jawbone has been traditionally considered palliative rather than curative, as it has been offered primarily to patients with advanced disease who have not responded to nonsurgical treatment [7, 17]. Gingival mucosa is known to not scar over a necrotic and exposed bone [18]. As therapeutic modalities for our patient, we opted radical approaches. The surgery was to be as conservative as possible, with a resection of the mandibular range followed by reconstruction using titanium plate with space maintainer. Some authors recommended the introduction of free flap bone [12]. However, most patients will not tolerate a long intervention with reconstruction because of their advanced age often associated with poor general condition [12]. The only risk to be expected is the plate’s exposure. This complication is the result of long-term friction between the plate and soft tissues [19]. A limitation of this case is that the luck of radiological examination (scan and orthopantomogram-OTP) because of the patient’s behavior (patient is quick-tempered, grumpy, and agitated).

## Conclusions

This case report is a very rare complication and the only preventive therapy we should have is the awareness. Mutual exchange between the maxillofacial and medical specialist (oncologists, general practitioners, rheumatologists, gynecologists, endocrinologists, and hematologists) is required and provision of detailed information of BRONJ/MRONJ to patients are essential for its prevention.
